# Effects of Preovulatory hCG Administration on Follicular Dynamics, Reproductive Hemodynamics, Nitric Oxide, and VEGF Levels in Mares

**DOI:** 10.3390/vetsci13080770

**Published:** 2026-07-31

**Authors:** Elshymaa A. Abdelnaby, Abdulrhman K. Alhaider, Ibrahim A. Emam, Hassan S. Al Qarnin

**Affiliations:** 1 Department of Clinical Sciences, College of Veterinary Medicine, King Faisal University, P.O. Box 400, Hofuf 31982, Saudi Arabia; 2 Department of Surgery, Anesthesiology and Radiology, Faculty of Veterinary Medicine, Cairo University, Giza 12211, Egypt

**Keywords:** human chorionic gonadotropin, equine, follicular dynamics, blood flow, nitric oxide

## Abstract

This study aimed to determine the action of a single dose of human chorionic gonadotropin (hCG) on follicular dynamics, CL functionality, ovarian (OV.A) and uterine arteries (MUA) blood flow, hormones (estradiol [E2] and luteinizing hormone [LH]), nitric oxide (NO), and vascular endothelial growth factor (VEGF) levels in Thoroughbred mares. Animals were subjected to ultrasonography scanning, when the dominant follicle (F1) reached 35 mm in diameter, mares were injected with hCG (hCG group; *n* = 7) at hour 0, while others were injected with saline (control; *n* = 7). The examination was performed a those points at hours −24 h, −12 h, 0 h, 12 h, 24 h, 36 h, 48 h, 72 h, and 96 h. Blood sampling, ultrasound scanning, hormone assaying, and statistical analysis were performed. F1 was ruptured at 12 h after injection compared to 24 h in the control, with an elevation in its diameter (*p* ≤ 0.05) and a colored area surrounding F1. The amount of color Doppler area around CL and its diameter were increased (*p* ≤ 0.05) at 24 and 48 h after injection with an increase (*p* ≤ 0.05) in the ipsilateral uterine horn colored area from 6 to 36 h after injection. OV.A and MUA Doppler velocity parameters were elevated (*p* ≤ 0.05) at 24 h. Both NO and VEGF levels were elevated at 24 h after injection, with the elevation (*p* ≤ 0.05) of E2 occurring only before injection and then declining. hCG and LH levels were increased after injection at 12 and 24 h, respectively, with an interaction (*p* ≤ 0.05) effect. hCG may speed up and hasten the ovulation process by elevating follicular color amount and CL morphometry via elevating ovarian flow and uterine body total coloration via elevating uterine blood flow (declining Doppler indices) in mares.

## Simple Summary

Ovulation is also induced by human chorionic gonadotropin (hCG) independent of the pituitary–adrenal axis. Generally, the use of hCG could be more efficient than GnRH in equines due to its easier availability and its lower cost. This current study aimed to determine the action of a single dose of hCG on follicular dynamics, luteal (CL) functionality, ovarian (OV.A) and uterine arteries (MUA) blood flow, hormones (estradiol [E2] and luteinizing hormone [LH]), nitric oxide (NO), and vascular endothelial growth factor (VEGF) levels in Thoroughbred mares. Blood sampling, ultrasound scanning, hormone assaying, and statistical analysis were performed. Using hCG in mares acts to induce predictable ovulation, mimic LH surge, and align breeding times with high accuracy.

## 1. Introduction

Most protocols of synchronization use two doses of gonadotropin-releasing hormone (GnRH) combined with a single dose of prostaglandin (PGF_2_α) as the Ovsynch system [1,2], as the application of GnRH is primarily related to the induction of a luteinizing hormone (LH) surge resulting in ovulation [3].

Moreover, unlike GnRH, LH works for the induction of ovulation, which is accompanied by the negative feedback of progesterone (P4) levels [4]. Ovulation is also induced by human chorionic gonadotropin (hCG) independent of the pituitary–adrenal axis. Generally, the use of hCG could be more efficient than GnRH in equines due to its easier availability and its lower cost [5,6].

hCG binds to LH receptors and mimics LH activity, thereby inducing ovulation and luteinization in cows [7], sheep [8,9], sows [10], and mares [11,12,13]. The application of hCG in the treatment of cystic ovaries [14], and synchronization was studied in animals regarding dose, timing of administration, and species [15,16,17].

Many factors contributed to the success of hCG as an ovulation-inducing agent, such as follicular diameter, as 1500–3000 IU of hCG should be administered when the largest follicle diameter reaches 32–35 mm [11]. This follicular factor has been reported in mares treated with hCG and GnRH [18,19]. In addition, uterine edema with a cartwheel appearance is an important factor related to uterine echogenicity and ovarian steroid circulating levels [20], with an elevation of estradiol (E2) levels that is critical in the release of LH and the induction of ovulation [21]. It was reported that treatment with hCG could result in a decline in E2 levels [22], but it resulted in a marked elevation of LH within 24 h in mares [23], in addition to the follicular diameter and uterine edema [24]. Another important parameter should be reported, which is genital blood flow. However, to the best of our knowledge, there is little data linking hCG injections and ovarian artery (OV.A) combined with middle uterine artery (MUA) blood flow. The vascular remodeling in mares could aid in reflecting the active physiological angiogenesis that is required for follicle maturation and successful implantation in relation to angiogenic factors [25,26]. The changes in blood flow may be associated with nitric oxide (NO) and vascular endothelial growth factor (VEGF) levels, as NO works in combination with E2 to increase both ovarian and uterine blood flow by the vasodilation process with upregulation of endothelial NO synthase type (eNOS) and thus leads to the relaxation of smooth muscle [26]. In addition, E2 enhances NO production and synthesis in uterine arteries that induce hyperemia during the estrous phase [27,28]. While VEGF is involved in the angiogenesis process with newly formed blood vessels and in the elevation of vascular permeability during equine follicular growth, ovulation, and formation of CL by facilitating follicular maturation and elevating blood supply to the ovaries by ovulation signals [29,30]. This study aimed to investigate the actions of a single administration of hCG on ovulation, ovarian and uterine blood flow patterns, hormonal levels (E2 and LH), and angiogenesis (NO and VEGF) processes in Thoroughbred mares.

## 2. Materials and Methods

### 2.1. Animals, Location and Management System

Thoroughbred multiparous mares (*n* = 14) were kept and handled at the College of Veterinary Medicine, King Faisal University—with a location (25°23′ N 49°36′ E) in ALHASA, Kingdom of Saudi Arabia, after obtaining acceptance and approval from the animal ethical committee at the Deanship of Ethical Review at King Faisal University (KFU-REC-2026-APR-ETHICS4170). Animals weighed about 500 ± 20 kg, aged about 6–10 years, and had a body condition score of about 4 [31]. Mares were non-lactating, and the interval from parturition to the first ovulation (partum-ovulation interval) typically averages 14 ± 4 days.

All animals were kept in an indoor paddock with the presence of a pubertal colt at the same stable to confirm and speed up estrous appearance behavior. Natural light was present all day, with about 12 h of artificial light at night within the paddock. Animals were fed a commercial pelleted ration for equines with hay, mineralized salt, and water during the day. All ultrasonographic scanning was performed by only one operator.

### 2.2. Experimental Design

**Figure 1:**
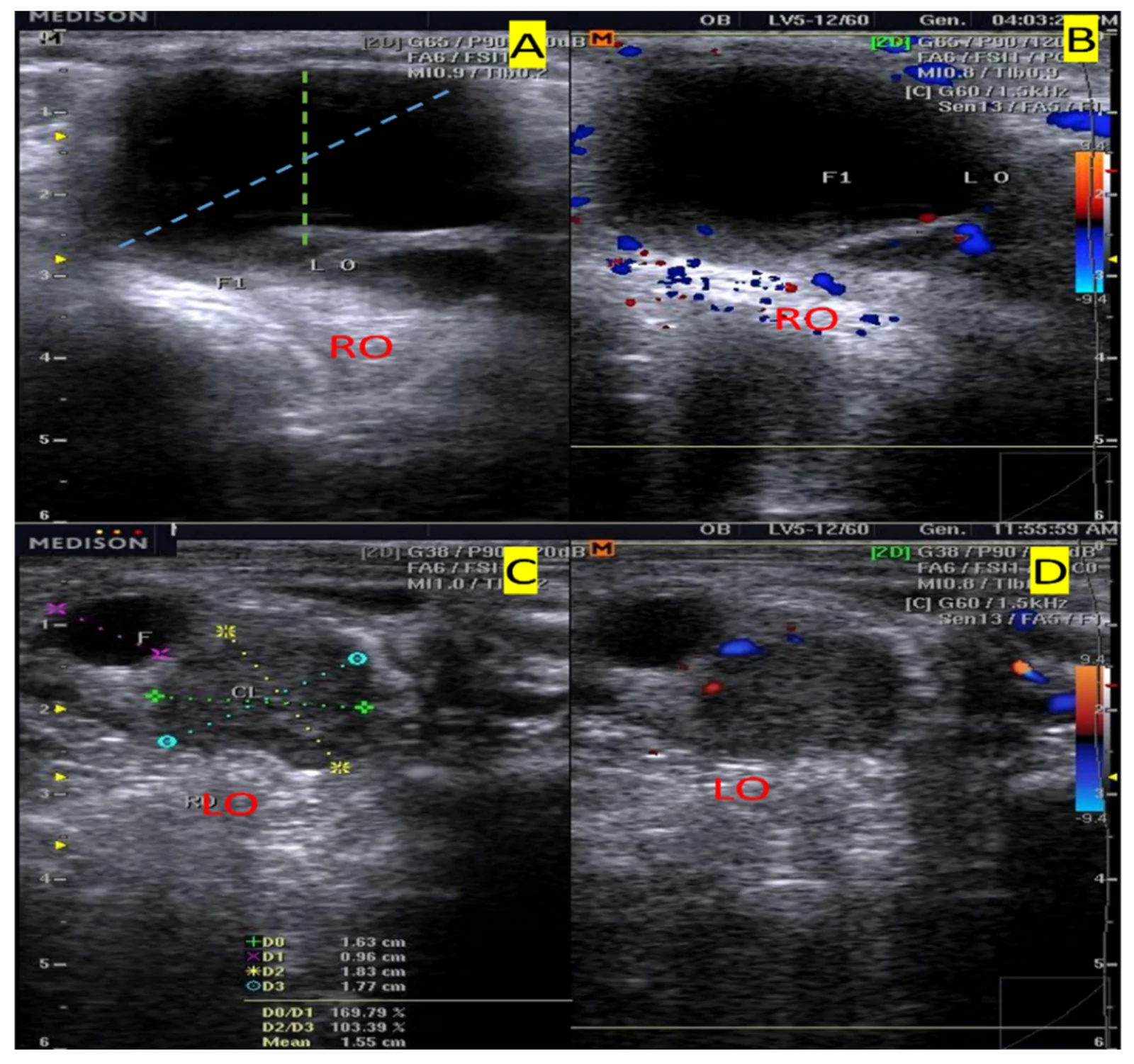
Ultrasonogram of the right ovary showed a dominant follicle with a diameter of 35.3 mm in B-mode gray scale (**A**), and the same follicle with color Doppler mode (**B**) after 6 h of hCG injection. In addition to the presence of left ovarian structures, such as corpus luteum and subordinate follicle with diameters of 11.65 and 9.88 mm, respectively, in B-mode gray scale (**C**), and the same follicle with luteal tissue in color Doppler mode (**D**). RO = right ovary, LO = left ovary, F1 = dominant largest follicle, F2 = subordinate follicle, and CL = corpus luteum.

**Figure 3:**
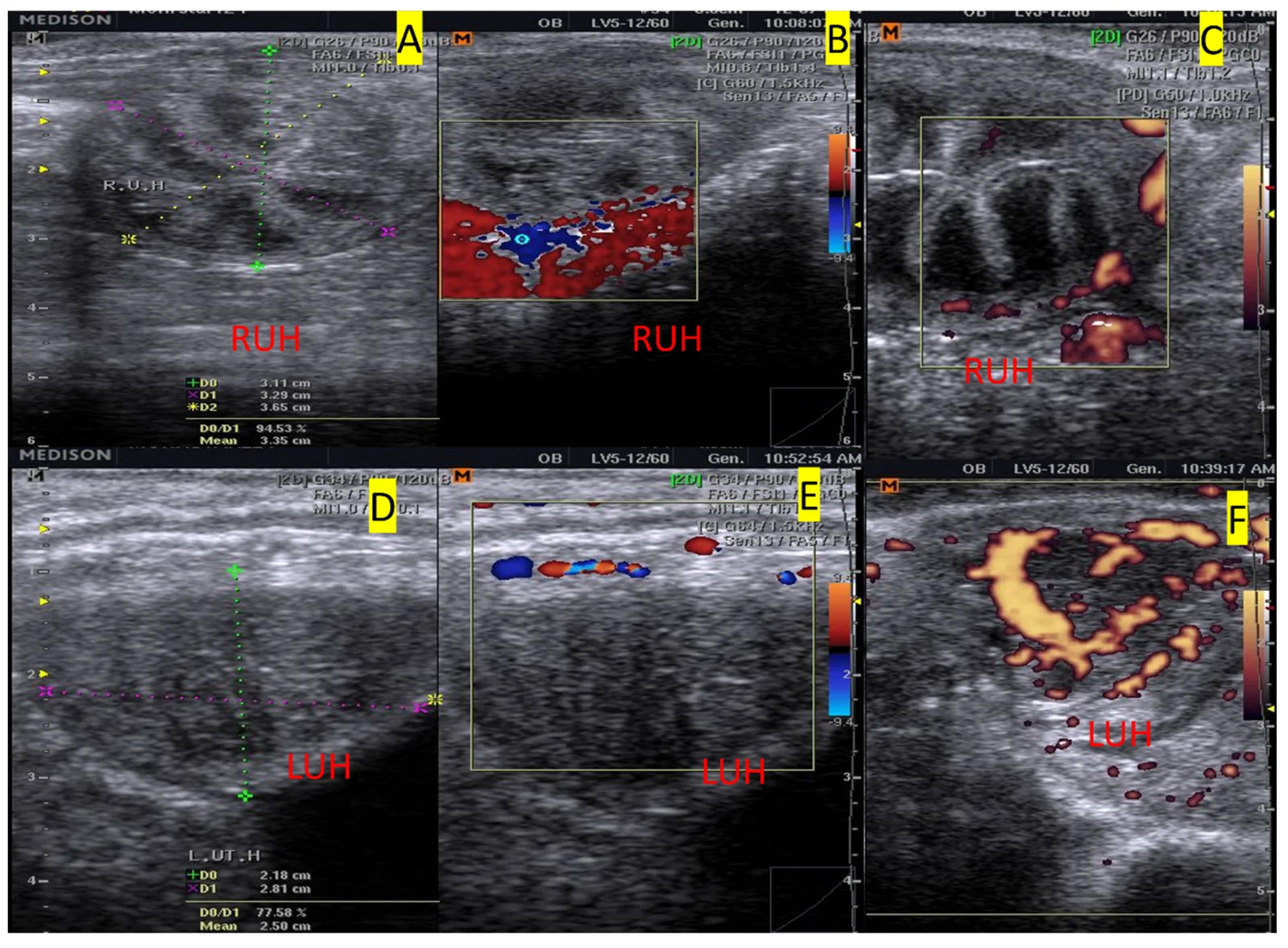
Ultrasonogram showed the cross-sectional diameter of the right uterine horn with B-mode (**A**) with appearance of cartwheel with a follicle 35.3 mm after 6 h of hCG injection, and colored area around the same horn with color mode (**B**) and power mode (**C**). in addition, left uterine horn was also measured in B-mode (**D**), and its coloration was estimated by color (**E**), and power (**F**) modes. RUH = right uterine horn, and LUH = left uterine horn.

This study was conducted from Jan 2026 to April 2026, as all mares must be examined for two successive estrous cycles to confirm animal functionality and ovarian cyclicity. All mares remained in good health with normal heat function during the study. Therefore, our inclusion criteria were the presence of normal healthy cyclic females in the proestrous phase, and that the females had not been injected with hCG during the previous year, and exclusion criteria were postpartum uterine infections (endometritis) and anatomical conformation issues (pneumovagina). At the third estrous cycle, a daily transrectal ultrasonography scan (MEDISION, SONOACEX8; 17 LCD MONITOR; Samsung Medison Co., Seoul, Replubic of Korea) was performed for all animals starting from 15 days after ovulation. Daily measurements of follicular diameters were conducted with an electronic caliper. When the largest dominant follicle reaches a diameter of 35 mm with the appearance of a cartwheel endometrial echogenic feature [32,33] as determined in Figure 1 and Figure 3, treated mares (hCG-treated group; *n* = 7) were subjected to a single intravenous injection of hCG (2500 IU; Ovogest; hCG; intravenous) at hour 0, and the same amount was injected into control mares in the form of saline (2.5 mL; control; *n* = 7) as used in a previous study [34]. The examination was performed at those points at hours (−24 h, −12 h, 0 h, 12 h, 24 h, 36 h, 48 h, 72 h, and 96 h). Ovaries were scanned every 12 h after injection to confirm ovulation, as the actual ovulation could occur between the detected ovulation and the previous scanning.

### 2.3. Ultrasonography Scanning

**Figure 2:**
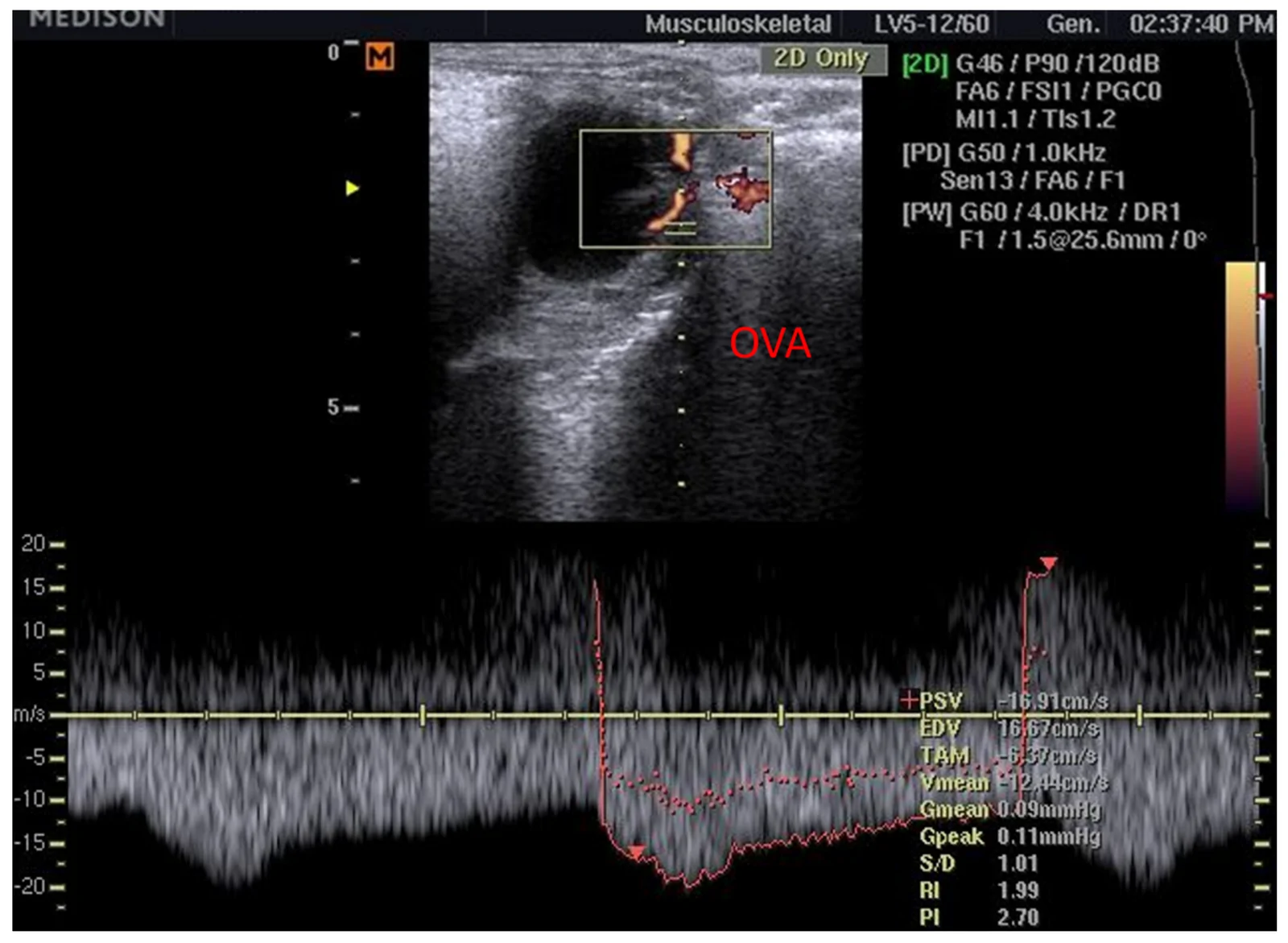
Ultrasonogram showed the largest follicle in a power mode Doppler to reveal a very small ovarian arterial supply and then a pulsed wave Doppler mode was activated to measure the three successive cardiac cycles to show Doppler parameter variations after 6 h of hCG injection. OVA = ovarian artery, PSV = peak systolic point of velocity (cm/s), EDV = end diastolic point of velocity (cm/s), TAM = time average maximum velocity to complete cycle (cm/s), S/D = ratio between systolic/diastolic, RI = resistance index of ovarian artery, and PI = pulsatility index of ovarian artery.

Both follicular and luteal tissue scanning was done by a transrectal probe equipped with a 5–12 MHz frequency for all mares (MEDISION, SONOACEX8; 17 LCD MONITOR; Samsung Medison Co., Seoul, Replubic of Korea). The device is equipped with Doppler modes (color, power, and pulsed waves). Dominant and subordinate follicle diameters were measured by an electronic caliper regarding corpus luteum (CL) diameter (Figure 1) after activation of the gray mode of scanning. Once color mode was activated, a total colored area surrounding the largest dominant follicle (F1) was estimated by two coloring maps (red and blue), and then power Doppler was activated to show a small branch of the ovarian artery (OVA) (Figure 2).

**Figure 4:**
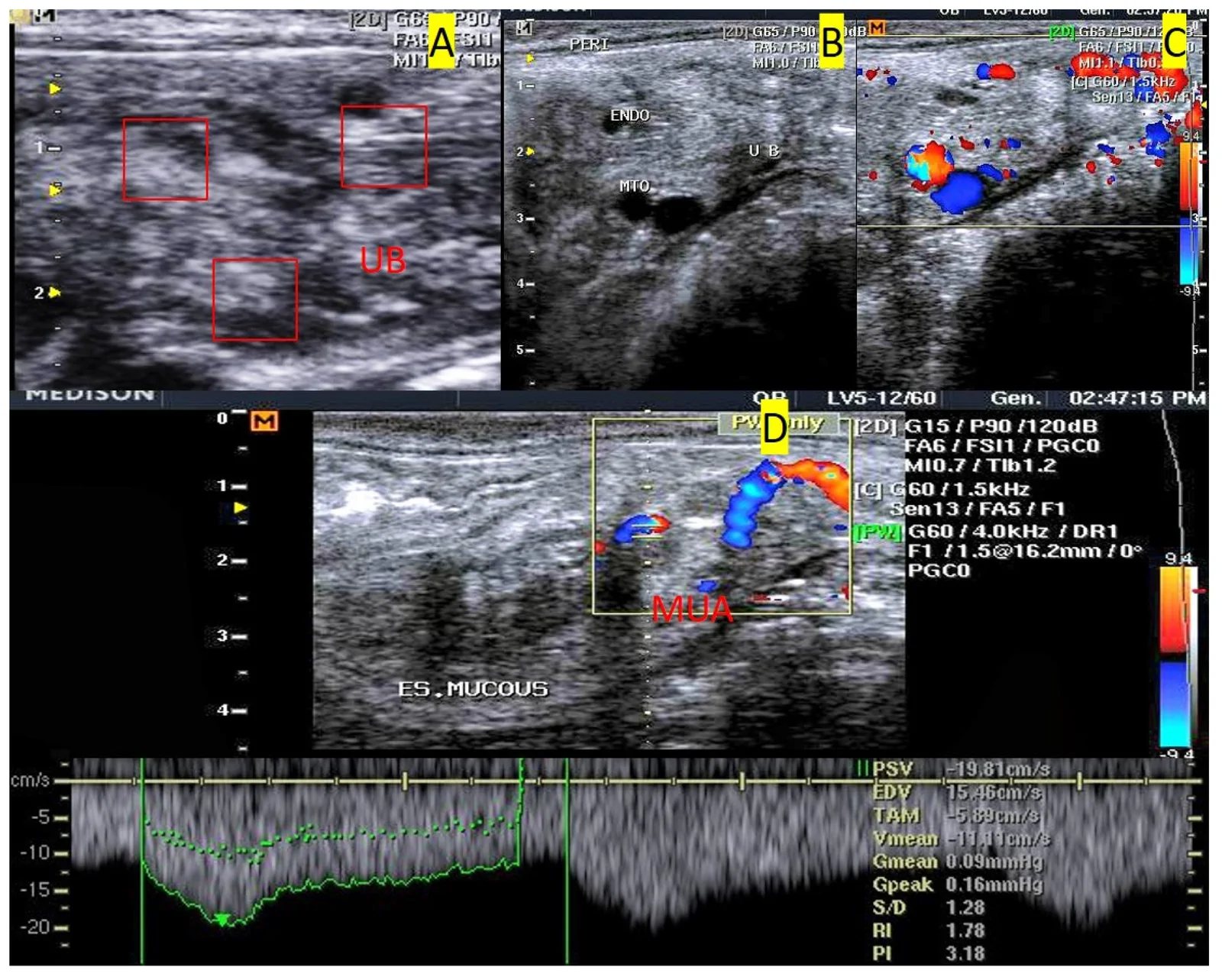
Ultrasonogram showed the drawing of three square (1 cm^2^) in uterine body to measure uterine tissue echogenicity (**A**), with a marked appearance of uterine layers (**B**), and its vascularization with coloration by color mode (**C**), in addition to middle uterine artery alterations by spectral Doppler mode to measure the three successive cardiac cycles to show Doppler parameters variations after 6 h of hCG injection (**D**). MUA = middle uterine artery, PSV = peak systolic point of velocity (cm/s), EDV = end diastolic point of velocity (cm/s), TAM = time average maximum velocity to complete cycle (cm/s), S/D = ratio between systolic/diastolic, RI = resistance index of middle uterine artery, PI = pulsatility index of middle uterine artery, ES mucous = estrus mucous, and UB = uterine body.

The colored area around the follicle was estimated by making an outline around the anechoic antral cavity as previously performed in mares [35]. The frozen colored image was transferred to the Adobe Photoshop program (1990–2013, Adobe Systems) to count the colored area with my magnetic lasso tool. Right and left uterine horn diameters and their colored area by color and power modes were calculated during the estimated time points (Figure 3). In addition, the uterine body was analyzed via uterine echotexture in the form of uterine tissue echogenicity (UE; NPVs) to estimate organ echotexture as previously determined [36] via a frozen B-mode ultrasonographic image (Figure 4). The uterine body colored area (UBCA; pixels; pixels) was estimated by the program (1990–2013, Adobe Systems), where the colored area was counted with my magnetic lasso tool. Finally, a window enters the gate of the known middle uterine artery (MUA) to measure the arterial waveform alterations (Figure 4). The Doppler parameters were automatically calculated once the operator got three successive waves with clear systolic and diastolic points, as those Doppler parameters were peak systolic point of velocity (PSV; cm/s), end diastolic point of velocity (EDV; cm/s), time average maximum velocity to complete cycle (TAM; cm/s), ratio between systolic/diastolic (S/D), resistance index (RI), and pulsatility index (PI) of both OVA and MUA. The settings of the B-mode and Doppler mode of an ultrasound scanning device were as follows: 3500 KHz as a pulse repetition frequency, 40° as an insonation angle, 0.5 mm as a gate window, 65 dB as gain, and two color maps [37,38,39].

### 2.4. Blood Collection and Hormonal Evaluation

Blood sampling was performed for all mares via the jugular vein. After blood collection, serum and plasma samples were obtained by centrifugation of whole blood samples at 1500× *g* for 15 min. All samples were stored at −18 °C until hormonal assaying. Plasma levels of hCG (MIU/mL) were assayed by radioimmunoassay with a two-site assay kit with antibody-coated V-tubes and 125^I^ detection labeling (Diagnostic Laboratories Science; DL 8350; Garden Grove, CA, USA). Plasma levels of luteinizing hormone (LH; ng/mL) were measured via radioimmunoassay with a presence of cross-reactivity between hCG and eLH with a 0.04%. All analyses were performed in our laboratory as previously done [33]. The test inter- and intra-assay coefficients of variance (CV %) were 4.9% and 5.1% for LH, with a sensitivity test of 0.2 ng/mL [11]. Regarding estradiol levels (E2; pg/mL), their plasma levels were determined by radioimmunoassay (double antibody kit; E2; Diagnostic Laboratories Science; Garden Grove, CA, USA). The inter- and intra-assay coefficients of variance (CV %) of E2 were 11.9% and 11.2%, with a sensitivity test of 0.2 pg/mL [11].

Nitric oxide levels (NO; µmol/L) were assayed via serum samples using diagnostic kits (R&D Systems, Inc., 614 McKinley Place, Minneapolis, MN, USA) with a catalog number DE1500B. The test inter- and intra-assay coefficients of variance (CV %) were 2.9% and 1.7%, with a sensitivity of 0.22 µmol/L [40]. Finally, vascular endothelial growth factor (VEGF; ng/mL) serum levels were calculated with double sandwich ELISA (MTS-1123-HM726; SUITE 203, 17 Ramsey Road, Shirley, NY 11967, USA) with a sensitivity of 18.77 ng/mL, an inter-assay coefficient of variance of 5.5%, and an intra-assay one of 4.5% [41].

### 2.5. Statistical Analysis

Data were represented as mean ± error of mean for all variables. All data must be firstly check and confirm for normality and homogeneity using the Kolmogorov–Smirnov test. A statistical analysis of all data was performed by SPSS software analysis program (Guide 20.0 Multilingual). Data were analyzed using mixed repeated-measures ANOVA with specific time points. Time effect (hour) and treatment effect (hCG-treated versus normal control mares) were measured. Tukey’s post hoc was done to estimate error bars in figures and the significance was set at a probability less than or equal to 0.05.

## 3. Results

### 3.1. Ultrasonographic Findings of the Genital Tract

#### 3.1.1. Effect of hCG on Follicular Dynamics, Corpus Luteum, and Uterine Horn Coloration

**Figure 5:**
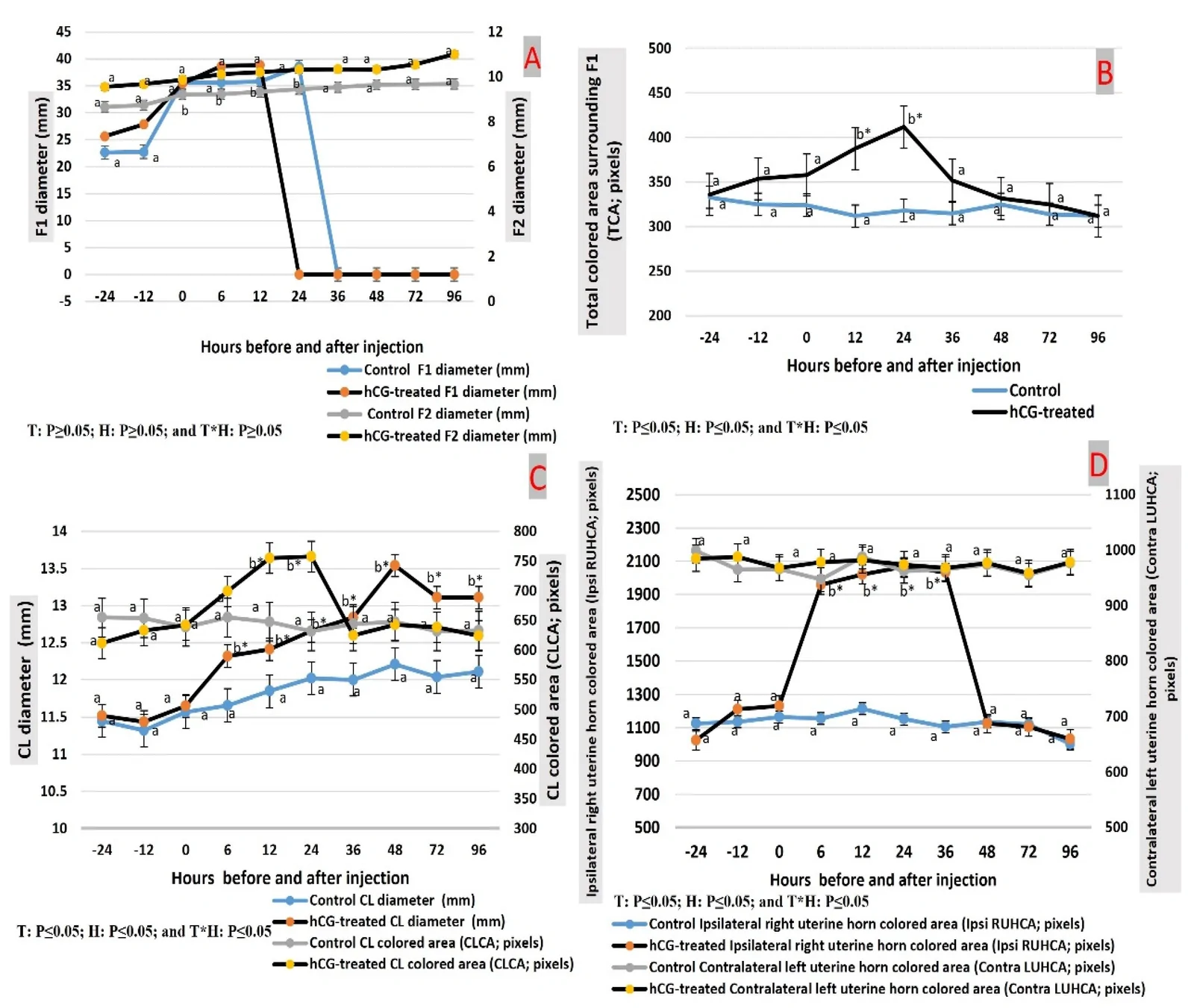
Mean ± standard error of mean (SEM) of the largest mature follicle diameter (F1 diameter; mm; primary axis; (**A**), and subordinate follicle diameter (F2 diameter; mm; (**A**); secondary axis), total colored area surrounding largest follicle (TCA; pixels; (**B**), corpus luteum diameter (CL diameter; mm; (**C**); primary axis), with its colored area (CLCA; pixels; (**C**); secondary axis), ipsilateral right uterine horn colored area (Ipsi RUHCA; pixels; (**D**); primary axis) and contralateral side (Contra LUHCA; pixels; (**D**); secondary axis) in control and hCG-treated females before and after hCG injection. Different superscript values indicate a significant difference (*p* ≤0.05) within groups at different hours, while * indicates a significant difference (*p* ≤ 0.05) between two groups at the same time.

The largest mature follicle diameter (F1) was increased (*p* ≤ 0.05) in hCG-injected females until 12 h after injection, then ruptured, while in control animals, F1 diameter was increased until 24 h after injection, then disappeared. Regarding the subordinate follicle (F2), its diameter was slightly increased but not significantly. There was a time effect only in F1 diameter levels between the two groups (*p* ≤ 0.05), as shown in Figure 5A. The total colored area surrounding the largest follicle (TCA; pixels) increased (*p* ≤ 0.05) after injection and reached its peak at 24 h compared to levels before injection and compared to the control (Figure 5B). There was a treatment effect with time and their interactions (*p* ≤ 0.05).

Both corpus luteum diameter and colored area (CL diameter: mm; Figure 5C, primary axis and CLCA: pixels; Figure 5C, secondary axis) were increased after injection, reaching a peak at 48 h and 24 h, respectively. In addition, the treatment effect (*p* ≤ 0.05), the hour effect (*p* ≤ 0.05), and their interaction (*p* ≤ 0.05) were observed during the hours of examination. The contralateral left side colored area of the uterine horn (Contra LUHCA; pixels; Figure 5D) showed no changes before and after hCG injection, while the ipsilateral right one (Ipsi RUHCA; pixels; Figure 5D) was increased (*p* ≤ 0.05) from 6 to 36 h after injection in hCG-treated females compared to controls.

#### 3.1.2. Effect of hCG on Uterine Echotexture and Uterine Body Coloration

**Figure 6:**
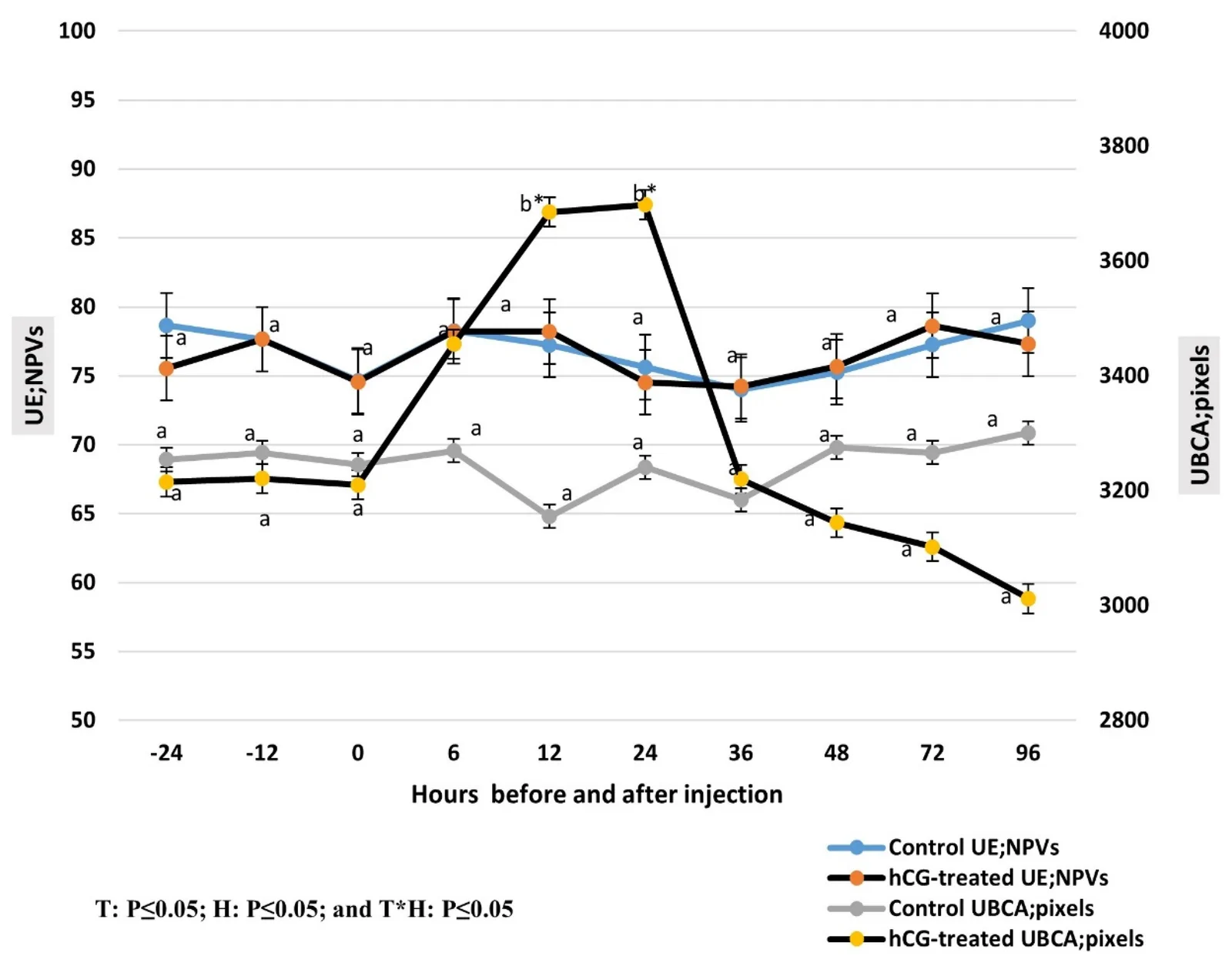
Mean ± standard error of mean (SEM) of the uterine echogenicity (UE; NPVs; primary axis), and uterine body colored area (UBCA; pixels; secondary axis) in control and hCG-treated females before and after hCG injection. Different superscript values indicate a significant difference (*p* ≤ 0.05) within groups at different hours, while * indicates a significant difference (*p* ≤ 0.05) between two groups at the same time.

Uterine tissue echotexture was measured by frozen ultrasonographic image in both groups. As depicted in Figure 6, uterine echogenicity (UE; NPVs; primary axis) was not altered (*p* > 0.05) by hCG injection before and after injection, while the uterine body colored area (UBCA; pixels; secondary axis; Figure 6) was elevated (*p* ≤ 0.05) until its peak at 24 h after injection in injected females compared to control females with time effect (*p* ≤ 0.05) and their interaction effect (*p* ≤ 0.05).

#### 3.1.3. Effect of hCG on Ovarian Artery and Middle Uterine Artery Flow

**Figure 7:**
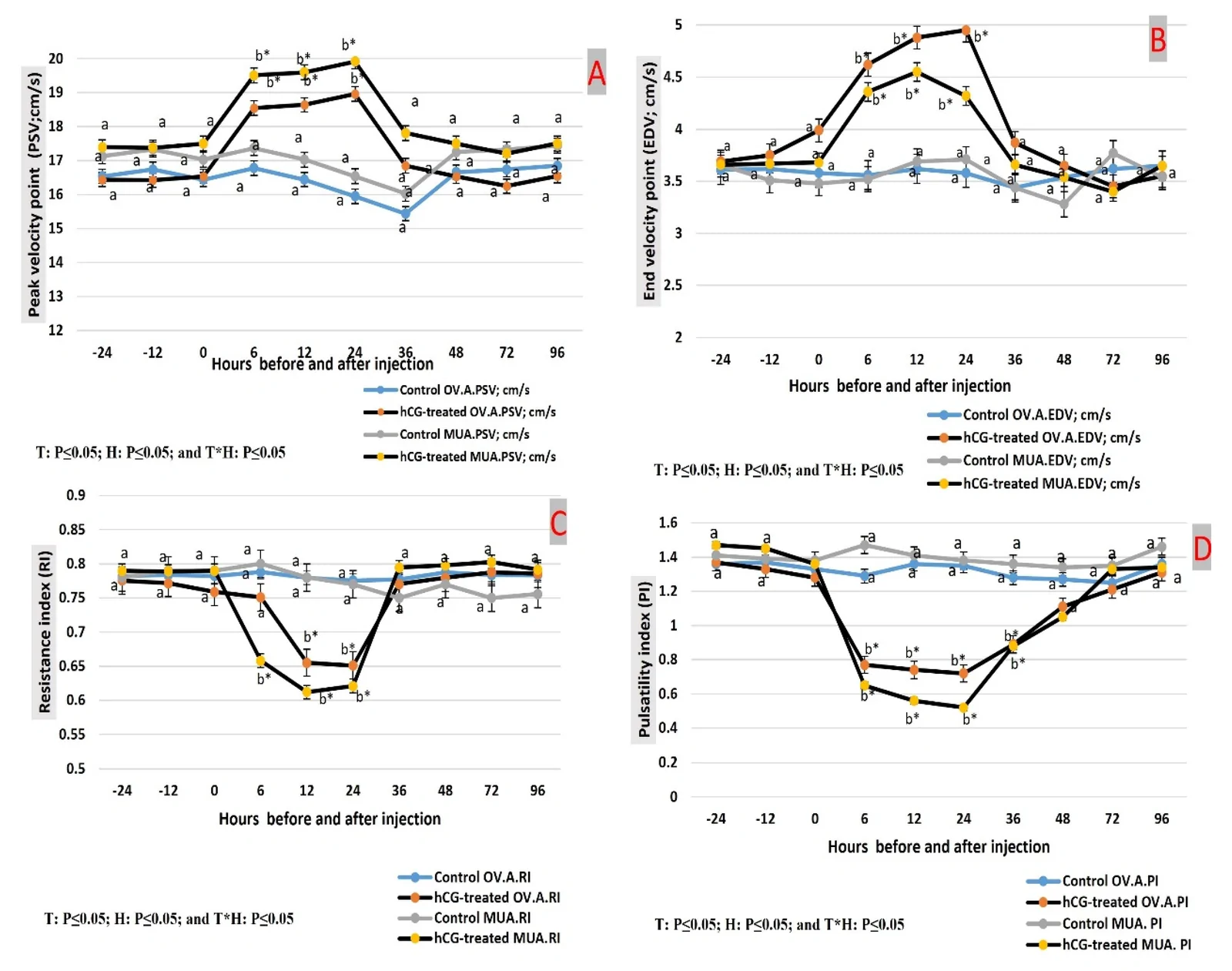
Mean ± standard error of mean (SEM) of the peak velocity point (PSV; cm/s; (**A**)), end velocity point (EDV; cm/s; (**B**)), resistance index (RI; (**C**)), and pulsatility index (PI; (**D**)) of both middle uterine and ovarian (MUA and OV. A) arteries in control and hCG-treated females before and after hCG injection. Different superscript values indicate a significant difference (*p* ≤ 0.05) within groups at different hours, while * indicates a significant difference (*p* ≤ 0.05) between two groups at the same time.

There was an hCG effect (*p* ≤ 0.05), an hour effect (*p* ≤ 0.05), and a treatment versus hour interaction effect (*p* ≤ 0.05) regarding alterations in blood flow velocities. The peak velocity point (PSV; cm/s) of ovarian and middle uterine arteries (OV. A and MUA) was increased (*p* ≤ 0.05) after injection, reaching the highest level at 24 h compared to before injection and control, as depicted in Figure 7A. While the end velocity point (EDV; cm/s; Figure 7B) of both arteries is increased (*p* ≤ 0.05), it reaches the highest level at 24 h for OV. A, and 12 h for MUA. Regarding both Doppler indices, both resistance (RI; Figure 7C) and pulsatility indices (PI; Figure 7D) of both arteries decreased (*p* ≤ 0.05) linearly after injection by 12 h for RI and by 24 h for PI. There was a treatment effect, a time effect, and their interactions (*p* ≤ 0.05).

### 3.2. Hormonal, Nitric Oxide, and Vascular Endothelial Growth Changes Following hCG Administration

**Figure 8:**
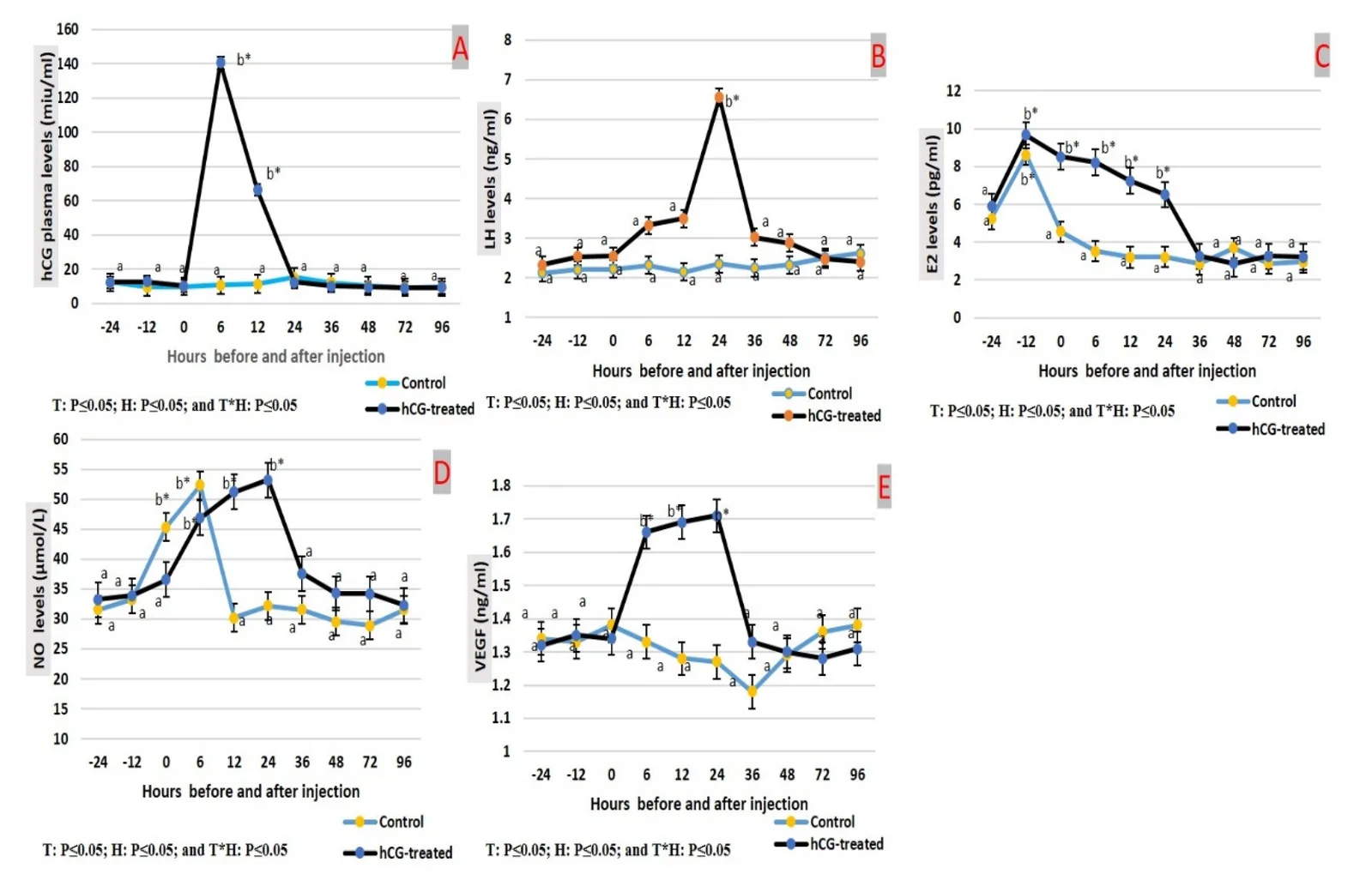
Mean ± standard error of human chorionic gonadotropin plasma levels (hCG; MIU/mL; (**A**)), luteinizing hormone (LH; ng/mL; (**B**)), estradiol levels (E2;pg/mL; (**C**)), nitric oxide levels (NO;µmol/L; (**D**)) and vascular endothelial growth factor (VEGF; ng/mL; (**E**)) levels in control and hCG-treated females before and after hCG injection. Different superscript values indicate a significant difference (*p* ≤ 0.05) within groups at different hours, while * indicates a significant difference (*p* ≤ 0.05) between two groups at the same time.

The plasma level of hCG (MIU/mL; Figure 8A) increased (*p* ≤ 0.05) by 12 h after injection but reached its peak at 6 h with treatment, time, and interactions. While luteinizing hormone (LH; ng/mL; Figure 8B) level was increased (*p* ≤ 0.05) after injection, it reached its peak at 24 h and then declined again until the end of the examination. E2 (pg/mL; Figure 8C) levels increased (*p* ≤ 0.05) before injection by 12 h and then remained at the same level of elevation until 24 h after injection. Regarding nitric oxide levels (NO; µmol/L; Figure 8D) and vascular endothelial growth factor (VEGF; ng/mL; Figure 8E) levels, both were elevated (*p* ≤ 0.05) after injection until they reached the highest levels at 24 h compared to before injection and to controls with treatment (*p* ≤ 0.05), time (*p* ≤ 0.05), and their interaction (*p* ≤ 0.05) effect, while NO levels were elevated in control group at the time of ovulation (*p* ≤ 0.05).

## 4. Discussion

A common and efficient hormone injection used to stimulate ovulation in mares (usually as Chorulon) is hCG, which usually takes effect 24 to 48 h after treatment. To control a mare’s reproductive cycles, ovulation is regularly induced. When cyclic estrous mares with a dominant follicle ≥35 mm in diameter are treated with hCG, ovulation will occur within 24 to 48 h. This study reported that earlier ovulation occurs in hCG-administered females compared with the control non-injected group. In treated females, F1 was present until 12 h and then disappeared, whereas it remained longer until 24 h in the non-injected control group. F1 diameter was increased after hCG administration, while the subordinate follicle showed no change in our study, which is in agreement with a study reporting a higher diameter of F1 in response to hCG and a lower diameter when hCG was combined with prostaglandin [13,42]. Similarly, when animals were injected with GnRH, ovulation was reported when F1 was more than 34mm compared to the control mare [43].

The elevation of CL diameter and its colored area with a previous elevation of coloration surrounding the follicle before ovulation in this study could be explained by the elevation of ovarian arterial blood flow with an elevation of its peak and end velocity points that spectral Doppler could calculate inside each artery, as this is the direct action of hCG in the stimulation of the angiogenesis process, so hCG enhances the new blood vessel formation [44]. The change in follicular flow with ovarian blood flow could be related to the marked decline of both Doppler indices, as reported by a study [25] that was observed with an elevation of CL diameter with a decline of ovarian PI and RI as a response to early implantation [45].

The elevation of the colored area at the ipsilateral horn is closely associated with the middle uterine artery alterations, as MUA travels in the broad ligament to supply both uterine horns, and the elevation of coloration on the ipsilateral side is a direct response to the change occurring on the same side regarding ovulation and CL formation [46]. The lower the arterial resistance, the higher the blood flow to the genital organs, such as testes with the testicular artery [47,48,49], ovaries with the ovarian artery [50,51], and uterine hemodynamics [39,52]. This current study revealed that no change occurred in uterine tissue echogenicity under hCG action. Contrary, a study revealed that hCG could alter the echogenicity of uterine tissue by making it more hyper-echoic to prepare the uterus for the implantation process [53,54]. The marked elevation of plasma levels of hCG after injection after 6 h in our study was accompanied by the long biological half-life of hCG (2–6 h) which could be considered a potent LH analog as it stimulates progesterone and testosterone secretion when binding with receptors [55,56]. hCG and LH share structural homology and bind to the same LH/CG because they do not actually “act together,” but rather activate the same receptor [57,58]. It is well known that the alpha subunit is similar between hCG and gonadotropin, while about 50% of the beta subunit is similar between hCG and eCG. This subunit contains the same amino acid sequence between eLH and eCG [59,60], so there is a similar representation between hCG and LH. It was demonstrated that the ovulation induction properties of hCG could result directly from the mechanism of the LH surge [61,62]. The vascular remodeling that is reflected by angiogenic factors with elevation of Doppler velocity parameters could be related to ovarian maturation that affects theca vasculature, and elevation of VEGF that is critical in follicle growth and rupture [62].

In our current study E2 was elevated 12 h before hCG injection in both groups and remained elevated until 24 h after injection, then declined. As is well-known, E2 in mares elevates around two–threefold during the period of luteal regression and then reaches its peak before ovulation by 2 days, which agrees with our findings, and then its levels decline after ovulation after 2–4 days in normal cases [63,64]. The elevation of E2 for 24 h in the hCG-treated group could be explained by the mimic LH surge accompanied by hCG injection that stimulates follicular growth and development with a temporary elevation of E2 levels by direct action on LH receptors [65]. Therefore, the hCG injection elevates both LH and E2 due to its action as a stimulant of E2 production by follicles that predict the success of IVF outcomes [66]. hCG acts as an LH agonist because it binds to the same receptor (LH/CGR). Therefore, its biological effects resemble an LH surge. The connection between E2 and LH levels in control and treated groups could be directly related to hCG-induced ovulation [22,67,68]. In addition, a negative relationship could be noticed with the LH surge when estradiol was administered [11,67]. Contrary to our findings, a study reported an elevation in E2 levels after hCG injection in buffalo bulls [69].

In the control, normal mares subjected to spontaneous ovulation, NO levels were increased at the time of ovulation, and this could be directly explained by the function of NO as a key regulator for the rupture of the follicle and the luteinization process with the increase in intra-follicular NO levels to facilitate the ovulation process [70,71]. In the treated group, there was a more elevated level of NO compared to the control on the day of ovulation. This could suggest faster growth and development of the growing and mature follicles after administration of hCG. On the day of ovulation, mares treated with hCG and PG had a significantly higher NO level compared to the control group. This suggests that the ovulating follicles grow and mature faster after receiving hCG and luteolysis. NO is known to modulate steroidogenesis in granulosa cells in cattle and mares, as well as follicular angiogenesis and blood flow. It also regulates ovulation by controlling the production of steroid hormones, which could be directly connected with the role of NO in the steroidogenesis of the granulosa cell [72], follicular hemodynamics with blood flow patterns [28], and steroid hormone production [73]. Moreover, the NO system in equine reproduction is a new research area of interest, as NO is primarily involved in vascular alterations required for ovulation with the aid of E2 that acts as a vasodilator in association with an elevation of the preovulatory follicular response to hCG [71,74]. In addition the elevation in ovarian hemodynamics could be related to NO metabolites that alter smooth muscle reactivity to manage vascular tone with VEGF up regulation to stimulate emergency angiogenesis under low oxygen [75]. Regarding the elevation of VEGF levels in mares injected with hCG, this could be associated with the role of hCG in the reproductive system’s angiogenesis by direct secretion of VEGF [76]. In addition, VEGF is widely distributed in all tissues, especially in the endocrine gland with micro-vessels, as it is reported and classified as an angiogenic selective factor [77,78]. By injection of hCG, LH receptors were activated to stimulate the expression of VEGF-A via the NF-κB pathway, promoting follicular development and the ovulation process with an elevation of VEGF [79]. By activating LH receptors, hCG induces VEGF expression and production in granulosa cells, promoting vascularization and mediating physiological changes such as follicular maturation and ovulation [80,81]. Our study limitations were a small sample size of mares, lack of progesterone analysis, and lack of previous research on the topic that could affect findings.

### Future Research Aspects

Our future research will be focused on tracking how chronic antibody production affects follicular wall blood flow and oocyte maturation, particularly in aged mares over 15 years old. In addition to trials for combination hCG with GnRH analogs to show their effect on the speed of ovulation without harming luteal function or early pregnancy.

## 5. Conclusions

A single dose of hCG before ovulation can greatly improve blood flow to the ovaries and uterus, raise levels of luteinizing hormone, nitric oxide, and vascular endothelial growth factor, and lower estradiol levels and both arterial Doppler indices. Additionally, hCG may speed up ovulation. In addition, hCG could hasten the ovulation process with an excellent follicular vascularization, with an elevation of corpus luteum diameter and colored area with respect to an elevation of the ipsilateral horn’s colored area.

## Data Availability

No new data were generated for this article. All datasets conducted during the current study are included in this article.

## References

[1] Pursley J.R., Mee M.O., Wiltbank M.C. (1995). Synchronization of ovulation in dairy cows using PGF2alpha and GnRH. Theriogenology.

[2] Refaat D., Ali A., Aljibali A., Ali M. (2025). Efficacy of ovulation synchronization in Arabian mares using Ovsynch program: A closer look to its feasibility. Int. J. Vet. Sci..

[3] Newcombe J.R., Cuervo-Arango J. (2017). What Are the Options for Induction of Ovulation in the Mare in Europe? Buserelin as an Alternative to Human Chorionic Gonadotropin. J. Equine Vet. Sci..

[4] Ginther O.J., Gastal E.L., Gastal M.O., Beg M.A. (2008). Dynamics of the Equine Preovulatory Follicle and Periovulatory Hormones: What’s New?. J. Equine Vet. Sci..

[5] Bottrel M., Ortiz I., Hidalgo M., Díaz-Jiménez M., Pereira B., Consuegra C., Yousef M.S., Dorado J. (2022). Hormonal Management for the Induction of Luteolysis and Ovulation in Andalusian Jennies: Effect on Reproductive Performance, Embryo Quality and Recovery Rate. Animals.

[6] Blanchard T.L., Brinsko S.P., Rigby S.L. (2002). Effects of deslorelin or hCG administration on reproductive performance in first postpartum estrus mares. Theriogenology.

[7] McCue P., Hudson J.J., Bruemmer J.E., Squires E.L. Efficacy of hCG at inducing ovulation: A new look at an old issue. Proceedings of the 50th Annual Convention of the American Association of Equine Practitioners.

[8] Liu T.C., Ho C.T., Li K.P., Chang C.C., Chan J.P. (2019). Human chorionic gonadotropin (hCG)-induced ovulation occurs later but with equal occurrence in lactating dairy cows: Comparing hCG and gonadotropin-releasing hormone protocols. J. Reprod. Dev..

[9] Bruno-Galarraga M., Cano-Moreno V., Lago-Cruz B., Encinas T., Gonzalez-Bulnes A., Martinez-Ros P. (2021). The Use of hCG for Inducing Ovulation in Sheep Estrus Synchronization Impairs Ovulatory Follicle Growth and Fertility. Animals.

[10] Cox J.F., Carrasco A., Navarrete F., Bocic A., Saravia F., Dorado J. (2024). A Subovulatory Dose of Human Chorionic Gonadotropin (hCG) May Sustain Terminal Follicle Development and Reproductive Efficiency during Anestrus in Sheep. Animals.

[11] Nissen A.K., Lehn-Jensen H., Hyttel P., Greve T. (1995). Follicular development and ovulation in sows: Effect of hCG and GnRH treatment. Acta Vet. Scand..

[12] Ginther O.J., Beg M.A., Gastal E.L., Gastal M.O., Cooper D.A. (2009). Treatment with human chorionic gonadotropin (hCG) for ovulation induction is associated with an immediate 17beta-estradiol decrease and a more rapid LH increase in mares. Anim. Reprod. Sci..

[13] Köhne M., Kuhl J., Ille N., Erber R., Aurich C. (2014). Treatment with human chorionic gonadotrophin before ovulation increases progestin concentration in early equine pregnancies. Anim. Reprod. Sci..

[14] Alkhadrawy J.M.H., Aboelmaaty A.M., Abou-Ahmed M., Ghallab A.M. (2024). Effect of hCG and prostaglandin on ovarian, luteal development, and hormonal changes in embryo donor mares during the hot summer months in subtropics. Open Vet. J..

[15] Ono T., Takagi M., Kawashima C., Wijayagunawardane M.P.B., Vos P.L.A.M., Taniguchi M., Tanihara F., Otoi T. (2018). Comparative Effects of Different Dosages of hCG on Follicular Development in Postpartum Dairy Cows With Cystic Ovarian Follicles. Front. Vet. Sci..

[16] Giordano J.O., Wiltbank M.C., Guenther J.N., Ares M.S., Lopes G., Herlihy M.M., Fricke P.M. (2012). Effect of presynchronization with human chorionic gonadotropin or gonadotropin-releasing hormone 7 days before resynchronization of ovulation on fertility in lactating dairy cows. J. Dairy Sci..

[17] Alonso M.A., Silva L.A., Affonso F.J., Lemes K.M., Celeghini E.C.C., Lançoni R., Carvalho H.F., de Arruda R.P. (2019). Effect of hCG application at different moments of the estrous cycle on corpus luteum and uterine vascularization and serum progesterone concentration in mares. Anim. Reprod..

[18] Zhang J., Liu Y., Yang N., Sun S., Li X., Wu X. (2025). Effects of human chorionic gonadotropin on reproductive outcomes in estrus-synchronized ewes subjected to two different insemination methods. Vet. Res. Forum.

[19] Barbacini S., Zavaglia G., Gulden P., Marchi V., Necchi D. (2000). Retrospective study on the efficacy of hCG in an equine artificial insemination programme using frozen semen. Equine Vet. Educ..

[20] Camillo F., Pacini M., Panzani D., Vannozzi I., Rota A., Aria G. (2004). Clinical use of twice daily injections of buserelin acetate to induce ovulation in the mare. Vet. Res. Commun..

[21] Pelehach L.M., Greaves H.E., Porter M.B., Desvousges A., Sharp D.C. (2002). The role of estrogen and progesterone in the induction and dissipation of uterine edema in mares. Theriogenology.

[22] Oberhaus E.L., Thompson D.L., Foster B.A., Pinto C.R. (2018). Effects of combined estradiol-sulpiride treatment and follicle ablation on vernal transition in mares: Evaluation of plasma and follicular fluid hormones and Luteinizing hormone receptor gene expression. J. Equine Vet. Sci..

[23] Jacob J.C., Gastal E.L., Gastal M.O., Carvalho G.R., Beg M.A., Ginther O.J. (2009). Temporal relationships and repeatability of follicle diameters and hormone concentrations within individuals in mares. Reprod. Domest. Anim..

[24] Gastal E.L., Silva L.A., Gastal M.O., Evans M.J. (2006). Effect of different doses of hCG on diameter of the preovulatory follicle and interval to ovulation in mares. Anim. Reprod. Sci..

[25] Cuervo-Arango J., Newcombe J.R. (2008). Repeatability of preovulatory follicular diameter and uterine edema pattern in two consecutive cycles in the mare and how they are influenced by ovulation inductors. Theriogenology.

[26] Bollwein H., Weber F., Kolberg B., Stolla R. (2002). Uterine and ovarian blood flow during the estrous cycle in mares. Theriogenology.

[27] Yilmaz Ö.T., Gündüz M.C., Evkuran Dal G., Uçmak M., Günay Uçmak Z., Karaçam E., Kaşikçi G., Kiliçarslan M.R. (2017). Evaluation of changes in Doppler ultrasonography indices and levels of maternal serum angiogenic factors throughout pregnancy in ewes. Theriogenology.

[28] Lechuga T.J., Qi Q.R., Kim T., Magness R.R., Chen D.B. (2019). E2β stimulates ovine uterine artery endothelial cell H2S production in vitro by estrogen receptor-dependent upregulation of cystathionine β-synthase and cystathionine γ-lyase expression. Biol. Reprod..

[29] Abdelnaby E.A., Alhaider A.K., El-Maaty A.M.A. (2023). Ovarian and uterine arteries blood flow velocities waveform, hormones and nitric oxide in relation to ovulation in cows superstimulated with equine chorionic gonadotropin and luteolysis induction 10 and 17 days after ovulation. BMC Vet. Res..

[30] Kaczmarek M.M., Schams D., Ziecik A.J. (2005). Role of vascular endothelial growth factor in ovarian physiology—An overview. Reprod. Biol..

[31] Gündüz M.C., Turna Ö., Uçmak M., Kaşıkçı G., Enginler S.Ö., Erzengin M., Kurban İ., Evkuran G., Akış I., Ateş A. (2014). Correlation between the serum and follicular fluid vascular endothelial growth factor A and nitric oxide levels and follicular vascularity in Arabian mares. J. Equine Vet. Sci..

[32] Fowler A.L., Pyles M.B., Bill V.T., Hayes S.H., Harris P.A., Lawrence L.M. (2020). Relationships Between Measurements of Body Fat in Thoroughbred Horses. J. Equine Vet. Sci..

[33] Ginther O.J., Pierson R.A. (1984). Ultrasonic anatomy and pathology of the equine uterus. Theriogenology.

[34] Duchamp G., Bour B., Combarnous Y., Palmer E. (1987). Alternative solutions to hCG induction of ovulation in the mare. J. Reprod. Fertil. Suppl..

[35] Abdelnaby E.A., Abo El-Maaty A.M., Ragab RSASeida A.A. (2016). Assessment of uterine vascular perfusion during the estrous cycle of mares in connection to circulating leptin, and nitric oxide concentrations. J. Equine Vet. Sci..

[36] Abdelnaby E.A., Abo El-Maaty A.M. (2017). Dynamics of Follicular Blood Flow, Antrum Growth, and Angiogenic Mediators in Mares From Deviation to Ovulation. J. Equine Vet. Sci..

[37] Demir M.C., Demir M.S., Büyükbaki B., Kuru M., Kaya S., Kaçar C. (2025). Changes in Doppler Ultrasonography and Echotexture Parameters in Cows During the Last 10 Days of Pregnancy. Vet. Med. Sci..

[38] Emam I.A., Alhaider A.K., Abdelnaby E.A. (2026). Effect of L-citrulline injection on testicular hemodynamics and semen quality in aged bucks. Vet. Res. Commun..

[39] Emam I.A., Alhaider A.K., Nahla M.A., Abdelnaby E.A. (2026). Effects of Sildenafil administration on placentome dynamics, uterine and umbilical blood flow patterns in relation to nitric oxide and vascular endothelial growth factor in advanced pregnant Hegazi goats. Theriogenology.

[40] Abdelnaby E.A., Alhaider A.K., Emam I.A. (2026). Repeated Melatonin Implant Improves Testicular Volume, Testicular Blood Flow, Estradiol, Antioxidant Capacity, and Nitric Oxide Levels in Post-Pubertal Camels Under Heat Stress Conditions. Reprod. Domest. Anim..

[41] Borunova S.F., Tkachev N., Iolchiev B., Artyushina Z., Abramov P., Nikitina M., Silanteva A., Khusnetdinova N., Serezhenkov V. (2020). Estimation of Nitrite-Nitric Oxide Derivative-In Horses with Intestinal Colic by ESR Spectroscopy. Vet. Sci..

[42] Schreurs M.P., Houston E.M., May V., Cipolla M.J. (2012). The adaptation of the blood-brain barrier to vascular endothelial growth factor and placental growth factor during pregnancy. FASEB J..

[43] Raz T., Hunter B., Carley S., Card C. (2009). Reproductive performance of donor mares subsequent to eFSH treatment in early vernal transition: Comparison between the first, second, and mid-season estrous cycles of the breeding season. Anim. Reprod. Sci..

[44] Stich K.L., Wendt K.M., Blanchard T.L., Brinsko S.P. (2004). Effects of a new injectable short-term release deslorelin in foal-heat mares. Theriogenology.

[45] d’Hauterive S.P., Close R., Gridelet V., Mawet M., Nisolle M., Geenen V. (2022). Human Chorionic Gonadotropin and Early Embryogenesis: Review. Int. J. Mol. Sci..

[46] Steier J.A., Bergsjø P.B., Thorsen T., Myking O.L. (2004). Human chorionic gonadotropin in maternal serum in relation to fetal gender and utero-placental blood flow. Acta Obstet. Gynecol. Scand..

[47] Cuervo-Arango J. (2025). Follicular growth, ovulation, and pregnancy responses to PGF-indued luteolysis and spontaneous return to estrus in Standardbred mares with large diestrous follicles. J. Equine Vet. Sci..

[48] Salama A., Abdelnaby E.A., Emam I.A., Fathi M. (2022). Single melatonin injection enhances the testicular artery hemodynamic, reproductive hormones, and semen parameters in German shepherd dogs. BMC Vet. Res..

[49] El-Sherbiny H.R., Hashem N.M., Abdelnaby E.A. (2023). Coat color affects the resilience against heat stress impacts on testicular hemodynamics, reproductive hormones, and semen quality in Baladi goats. BMC Vet. Res..

[50] Abdelnaby E.A. (2025). The impacts of L-citrulline and sildenafil on penile and testicular vascular perfusion pattern, advanced testicular analysis, steroids, and semen picture in normospermic dogs. Vet. Res. Commun..

[51] Abdelnaby E.A., Abo El-Maaty A.M., El-Badry D.A. (2020). Ovarian and uterine arteries blood flow waveform response in the first two cycles following superstimulation in cows. Reprod. Domest. Anim..

[52] Daghash S.M., Yasin N.A.E., Abdelnaby E.A., Emam I.A., Tolba A., Abouelela Y.S. (2022). Histological and hemodynamic characterization of corpus luteum throughout the luteal phase in pregnant and non-pregnant buffalos in relation to nitric oxide levels based on its anatomical determination. Front. Vet. Sci..

[53] Sprague B.J., Phernetton T.M., Magness R.R., Chesler N.C. (2009). The effects of the ovarian cycle and pregnancy on uterine vascular impedance and uterine artery mechanics. Eur. J. Obstet. Gynecol. Reprod. Biol..

[54] Fanchin R., Righini C., Ayoubi J.M., Olivennes F., de Ziegler D., Frydman R. (2000). New look at endometrial echogenicity: Objective computer-assisted measurements predict endometrial receptivity in in vitro fertilization-embryo transfer. Fertil. Steril..

[55] Hou Z., Zhang Q., Zhao J., Xu A., He A., Huang X., Xie S., Fu J., Xiao L., Li Y. (2019). Value of endometrial echo pattern transformation after hCG trigger in predicting IVF pregnancy outcome: A prospective cohort study. Reprod. Biol. Endocrinol..

[56] Ezcurra D., Humaidan P. (2014). A review of luteinising hormone and human chorionic gonadotropin when used in assisted reproductive technology. Reprod. Biol. Endocrinol..

[57] Gallelli M.F., Bianchi C., Zampini E., Aba M., Rodriguez M., Neild D., Carretero M.I., Miragaya M. (2025). Effect of hCG administration on corpus luteum development and plasma progesterone concentration in llamas. Theriogenology.

[58] Choi J., Smitz J. (2014). Luteinizing hormone and human chorionic gonadotropin: Distinguishing unique physiologic roles. Gynecol. Endocrinol..

[59] Lazzaretti C., Secco V., Paradiso E., Sperduti S., Rutz C., Kreuchwig A., Krause G., Simoni M., Casarini L. (2020). Identification of Key Receptor Residues Discriminating Human Chorionic Gonadotropin (hCG)- and Luteinizing Hormone (LH)-Specific Signaling. Int. J. Mol. Sci..

[60] Sullivan J.J., Parker W.G., Larson L.L. (1973). Duration of estrus and ovulation time in non lactating mares given hCG during three successive estrous periods. J. Am. Vet. Med. Assoc..

[61] Munshi U.M., Pogozheva I.D., Menon K.M. (2003). Highly conserved serine in the third transmembrane helix of the luteinizing hormone/human chorionic gonadotropin receptor regulates receptor activation. Biochemistry.

[62] Galet C., Chopineau M., Martinat N., Combarnous Y., Guillou F. (2000). Expression of an in vitro biologically active equine LH/CG without C-terminal peptide (CTP) and/or β26-110-disulphide bridge. J. Endocrin..

[63] Tamanini C., De Ambrogi M. (2004). Angiogenesis in developing follicle and corpus luteum. Reprod. Domest. Anim..

[64] Adams T.E., Horton M.B., Watson J., Adams B.M. (1986). Biological activity of luteinizlng hormone (LH) during the estrous cycle of mares. Domest. Anim. Endocrinol..

[65] Watson E., Al-Ziábi M. (2002). Characterization of morphology and angiogenesis in follicles of mares during spring transition and the breeding season. Reproduction.

[66] Malathi A., Balakrishnan S. (2021). Correlation between estradiol levels on day of HCG trigger and the number of mature follicles, number of oocytes retrieved, and the number of mature oocytes (M_2_) after oocyte aspiration in ICSI cycles. Middle East Fertil. Soc. J..

[67] Foroozanfard F., Moraveji S.A., Taghavi S.A., Karimi F. (2016). Association Between Serum Estradiol Level on the Day of hCG Administration and IVF-ICSI Outcome. J. Obs. Gynaecol. India.

[68] Ginther O.J., Utt M.D., Bergfelt D.R., Beg M.A. (2006). Controlling interrelationships between progesterone/LH and estradiol/LH during the equine estrous cycle. Anim. Reprod. Sci..

[69] Ginther O.J., Utt M.D., Beg M.A., Gastal E.L., Gastal M.O. (2007). Negative effect of estradiol on luteinizing hormone throughout the ovulatory luteinizing hormone surge in mares. Biol. Reprod..

[70] Abdelnaby E.A. (2022). Testicular haemodynamics, plasma testosterone and oestradiol concentrations, and serum nitric oxide levels in the Egyptian buffalo bull after a single administration of human chorionic gonadotropin. Reprod. Domest. Anim..

[71] Pinto C.R., Paccamonti D.L., Eilts B.E., Short C.R., Godke R.A. (2002). Effect of nitric oxide synthase inhabitors on ovulation in hCG-stimulated mares. Theriogenology.

[72] Pinto C.R., Paccamonti D.L., Eilts B.E., Venugopal C.S., Short C.R., Gentry L.R., Thompson D.L., Godke R.A. (2003). Concentrations of nitric oxide in equine preovulatory follicles before and after administration of human chorionic gonadotropin. Theriogenology.

[73] Matsumi H., Yano T., Osuga Y., Kugu K., Tang X., Xu J.P., Yano N., Kurashima Y., Ogura T., Tsutsumi O. (2000). Regulation of nitric oxide synthase to promote cytostasis in ovarian follicular development. Biol. Reprod..

[74] Huang H.F., Wang B., Yang X.F., Luo Q., Sheng J.Z. (2005). Nitric oxide mediates inhibitory effect of leptin on insulin-like growth factor I augmentation of 17β-estradiol production in human granulosa cells. Biol. Reprod..

[75] Parraguez V.H., Urquieta B., Pérez L., Castellaro G., De los Reyes M., Torres-Rovira L., Aguado-Martínez A., Astiz S., González-Bulnes A. (2013). Fertility in a high-altitude environment is compromised by luteal dysfunction: The relative roles of hypoxia and oxidative stress. Reprod. Biol. Endocrinol..

[76] Luo Y., Zhu Y., Basang W., Wang X., Li C., Zhou X. (2021). Roles of Nitric Oxide in the Regulation of Reproduction: A Review. Front. Endocrinol..

[77] Harada M., Peegel H., Menon K.M. (2010). Expression of vascular endothelial growth factor A during ligand-induced down-regulation of luteinizing hormone receptor in the ovary. Mol. Cell Endocrinol..

[78] LeCouter J., Kowalski J., Foster J., Hass P. (2001). Identification of an angiogenic mitogen selective for endocrine gland endothelium. Nature.

[79] LeCouter J., Lin R., Ferrara N. (2002). Endocrine gland-derived VEGF and emerging hypothesis of organspecific regulation of angiogenesis. Nat. Med..

[80] Ishikawa K., Ohba T., Tanaka N., Iqbal M., Okamura Y., Okamura H. (2003). Organ-specific production control of vascular endothelial growth factor in ovarian hyperstimulation syndrome-model rats. Endocr. J..

[81] Levitas E., Huleihal M., Lunenfeld E., Gakman R., Friger M., Potashnik G. (2013). Variations in vascular endothelial growth factor levels during ovarian superovulation and reduction of ovarian hyperstimulation incidence in young women: A prospective study. Open J. Obs. Gynecol..

